# iCAZyGFADB: an insect CAZyme and gene function annotation database

**DOI:** 10.1093/database/baad086

**Published:** 2023-11-28

**Authors:** Chun Fu, YaoJun Yang

**Affiliations:** Key Laboratory of Sichuan Province for Bamboo Pests Control and Resource Development, Leshan Normal University, No. 778 Binhe Road, Shizhong District, Leshan, Sichuan 614000, China; Key Laboratory of Sichuan Province for Bamboo Pests Control and Resource Development, Leshan Normal University, No. 778 Binhe Road, Shizhong District, Leshan, Sichuan 614000, China

## Abstract

With the continuous upgrading of high-throughput sequencing technology, a large amount of biological genome data has been deciphered and published. The research on functional genes of biological genomes urgently needs a collection of service websites with user-friendly and full annotation functions for a variety of gene function annotation tools. In this study, iCAZyGFADB, which is a database website integrating nine gene function annotation tools, was perfectly developed to meet the needs of biological genome functional annotation. Its nine gene function annotation tools were Carbohydrate-Active Enzymes (CAZyme) annotation, Gene Ontology (GO) annotation, Kyoto Encyclopedia of Genes and Genomes (KEGG) annotation, Cluster of Orthologous Gene (COG) annotation, Evolutionary Genealogy of Genes: Non-supervised Orthologous Groups (eggNOG) annotation, SwissProt annotation, Pfam annotation, KOG annotation and Animal Transcription Factor DataBase (AnimalTFDB) annotation. It has three advantages. First, it is superior to gene function annotation of other biological cloud analysis platforms and runs very fast. Second, all gene annotation functions of the website are free and open to users. Third, it can annotate eight gene functions (GO, KEGG, COG, eggNOG, SwissProt, Pfam, KOG and AnimalTFDB annotation) of a single species at the same time, while other cloud platforms do not have the ability or need to charge to open for users to complete the annotation of eight gene functions at the same time. Moreover, the development and operation of our database will provide great help for gene function annotation research and significantly improve the efficiency of genome function research and reduce the cost of bioinformatics analysis. Genomic functional annotation researchers can access this database through the following website: http://www.icazygfadb.org.cn/.

**Database URL:**  http://www.icazygfadb.org.cn/

## Introduction

The development of high-throughput sequencing technology has led to the explosive generation of genomic data, and the research of life science has entered the big data era. For example, as of 1 September 2022, the NCBI database (https://www.ncbi.nlm.nih.gov/) (1) has published 1033 plant genomes, 1802 insect genomes, 2046 archaea genomes, 3774 fungal genomes, 33 779 bacterial genomes, 595 avian genomes, 867 fish genomes and 677 mammalian genomes. Neither agriculture that is closely related to people’s lives nor medicine that is related to people’s health can be separated from the development and research of multiomics technology (2), especially the research of functional genes in the genome is primary, very important and very basic for crop breeding and medical research (3). However, the study of gene function in the genomes cannot be separated from functional gene annotation. In general, the functional annotation of genome refers to Gene Ontology (GO), Kyoto Encyclopedia of Genes and Genomes (KEGG), Clusters of Genes (COG), Evolutionary Genealogy of Genes: Non-supervised Orthologous Groups (eggNOG), SwissProt, Pfam, KOG, Carbohydrate-Active Enzymes (CAZyme) and AnimalTFDB annotation. The Gene Ontology resource (http://geneontology.org), founded in 1998, provides structured, computable knowledge regarding the functions of genes and gene products (4, 5). KEGG (http://www.genome.ad.jp/kegg/) is a knowledge base for systematic analysis of gene functions, linking genomic information with higher-order functional information (6). The COG database (https://www.ncbi.nlm.nih.gov/research/COG), also referred to as the Clusters of Orthologous Groups of proteins, was created in 1997 and went through several rounds of updates, most recently, in 2014 (7). EggNOG is a public database of orthology relationships, gene evolutionary histories and functional annotations (8). SwissProt’s (UniProtKB) (http://www.uniprot.org/) main goal is to provide the scientific community with a central resource for protein sequences and functional information (9). The Pfam database (http://pfam.xfam.org/) is a widely used resource for classifying protein sequences into families and domains (10). KOG databases (ftp://ftp.ncbi.nih.gov/pub/COG/KOG/kyva) revealed functional clusters associated with immunity and inflammation, oxidative stress, biosynthesis and metabolism (11). The CAZy (http://www.cazy.org) provides online and continuously updated access to a sequence-based family classification linking the sequence to the specificity and 3D structure of the enzymes that assemble, modify and breakdown oligo- and polysaccharides (12). The dbCAN2 (https://bcb.unl.edu/dbCAN2/) is presented as an updated metaserver, which integrates three state-of-the-art tools for CAZome (all CAZymes of a genome) annotation (13). The Animal Transcription Factor DataBase (AnimalTFDB) is a resource aimed to provide the most comprehensive and accurate information for animal transcription factors (TFs) and cofactors (14). The abovementioned nine genome annotation tools are designed separately and independent of different databases and websites. To annotate gene function of the genome data of a certain insect, the abovementioned nine gene function annotation tools can be used separately or simultaneously to annotate the functions of all genes in the species. The procedures required for genome function annotation are very complex, troublesome and time-consuming and cannot well meet the needs of genome function researchers, and the cost is high.

In the face of these difficulties, researchers of some bioinformation technology companies have developed some biological information cloud service platforms to meet the needs of researchers. For example, Biomarker Technologies Co., Ltd has developed a BMKcloud platform (https://international.biocloud.net/), in which there is a gene function annotation tool, including Go, KEGG, COG, SwissProt, TrEMBL, KOG and Pfam annotation. The gene function annotation tool in the BMKcloud platform can annotate the above seven functions of a biological genome at the same time. However, this gene function annotation tool is not free for users. Users need to pay a fee to become members before using it for free. A Omicshare cloud platform (https://www.omicshare.com/tools) developed by GENE DENOVO Biotechnology Co., Ltd contains 15 annotation tools, including GO, KEGG, PlantTFdb, NR, KOG, Pfam, COG, SwissProt, PHI, CAZy, eggNOG, VFDB, AnimalTFDB, CARD and ARDB annotation. Although the Omicshare cloud platform contains 10 gene annotation tools, each annotation tool is independent and can only be used separately. It cannot perform functional annotation analysis on a biological genome at the same time. In particular, it should be noted that these gene function annotation tools in the cloud platform are not free to users. Users can only use them after becoming a member and paying a certain fee.

In terms of functional insert olfactory receptor gene annotation, Li *et al.* have built the iORbase database (15). The iORbase database was used for functional insect olfactory receptor gene annotation, as it has a structural validation strategy that can greatly reduce false positives during functional OR gene annotation. Moreover, in the development and research of plant genome gene function annotation tools, Shen *et al.* have developed a GFAP web and software version of the gene function annotation tool (16). The web version of the gene annotation tool limits the number of species protein sequences, that is, the protein file cannot exceed 1 M, which greatly reduces the convenience and availability of annotation for all plant genome protein sequences. The GFAP annotation tool can only annotate the annotation function of GO, KEGG and Pfam simultaneously. Both gene function annotation tools of the cloud platform developed by the above companies and the web version of gene function annotation tools developed by some researchers are not yet comprehensive enough to integrate multiple gene annotation tools. There are also many limitations, such as restrictions on the number of protein sequence entries or protein file size of species, or the need to pay expensive service fees to use them.

To sum up, although powerful bioinformation technology companies have developed a cloud platform containing a variety of gene function annotation tools, which can provide paid services for genome researchers or users, the inconvenience and high cost of gene function annotation are still important factors restricting genome function researchers’ study. Therefore, iCAZyGFADB (http://www.icazygfadb.org.cn/), which has been developed by our team, is a comprehensive service tool integrating nine gene function annotation tools. It can be used to annotate nine gene functions of a certain biological genome at the same time and is user-friendly. It can be used to annotate gene functions of all insects’ genomes, which is best to be with annotated complete protein sequences.

## Materials and methods

### Data acquisition and source

iCAZyGFADB has completed annotation analysis of *CAZyme* gene family in 151 insect genomes. The genome sequences, protein sequences and annotation files of these 151 insect genomes are from NCBI (https://www.ncbi.nlm.nih.gov/) (1) and European biological information database (https://www.ebi.ac.uk/ena/browser/home) (Supplementary Table S1). The annotation comparison data used in the design of the CAZyme annotation tool comes from the dbCAN2 meta server (http://bcb.unl.edu/dbCAN2/).The annotation comparison data used in the design of GO, KEGG, COG, eggNOG, SwissProt, Pfam, KOG and AnimalTFDB annotation tool, respectively, comes from Gene Ontology Resource (http://geneontology.org/) (4–6), KEGG (https://www.kegg.jp/kegg/) (6), COG database (https://www.ncbi.nlm.nih.gov/research/cog-project/) (7), EggNOG (http://eggnog5.embl.de/#/app/home) (8), SwissProt database (https://www.uniprot.org/help/uniprotkb) (9), Pfam database (http://pfam.xfam.org/) (10) KOG (ftp://ftp.ncbi.nih.gov/pub/COG/KOG/) (11) and AnimalTFDB4.0 (http://bioinfo.life.hust.edu.cn/AnimalTFDB4/#/) (14).

### Gene function annotation design method

Nine gene function annotation tools are designed in insect CAZyme and Gene Function Annotation Database (iCAZyGFADB) developed by our team. Each tool can annotate the gene function of any insect genome. CAZyme annotation tools can annotate proteins together using HMMER (17, 18), DIAMOND (19) and Hotpep (20) via CAZy (12), dbCAN2 (13), CAZyme peptides, respectively. GO (4, 5), KEGG (6), COG (7), eggNOG (8), SwissProt (9), Pfam (10), KOG (11) and AnimalTFDB (14) annotation tools can annotate and analyze all protein sequences of insect genome alone or at the same time.

## Results

### Layout and content of iCAZyGFADB

**Figure 1.**
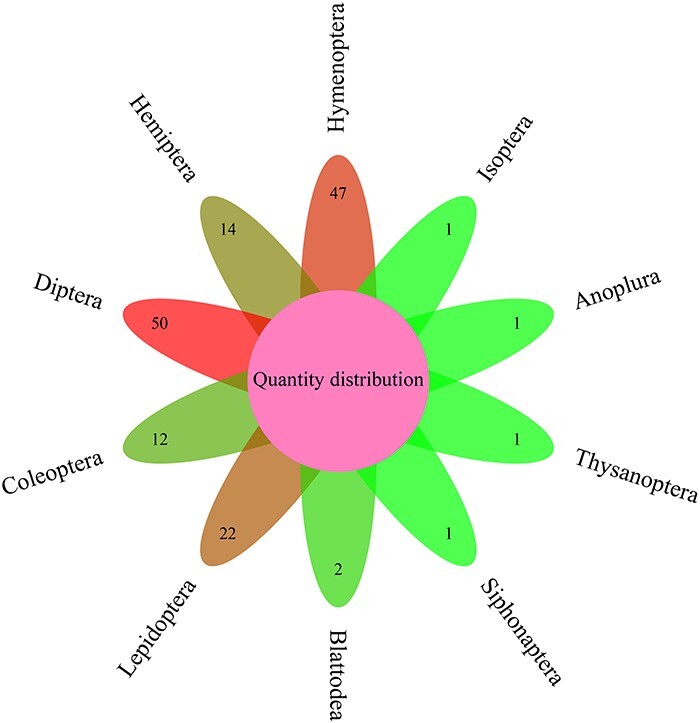
Distribution of insect numbers per order for 151 insects.

**Figure 2.**
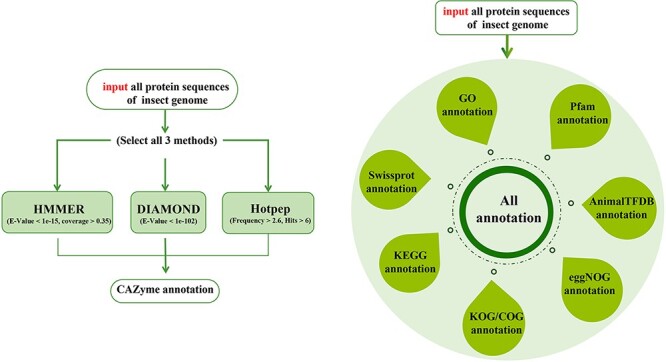
Design flow chart of iCAZyGFADB.

In order to facilitate annotation of functional genes in insect genomes and reduce their costs, this study constructed insect CAZyme and Gene Function Annotation Database (iCAZyGFADB). The number of 151 species of insects used for building iCAZyGFADB database is the largest in Diptera (50), followed by Hymenoptera (47) (Figure 1). This database is designed based on CAZyme annotations and eight gene functional annotations. The design method is shown in Figure 2.

**Figure 3.**
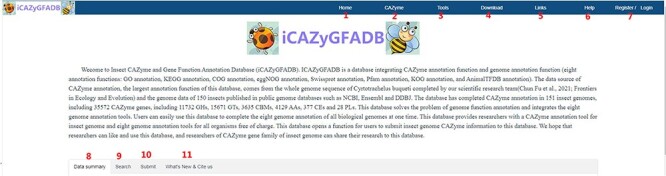
Content and layout of iCAZyGFADB.

**Figure 4.**
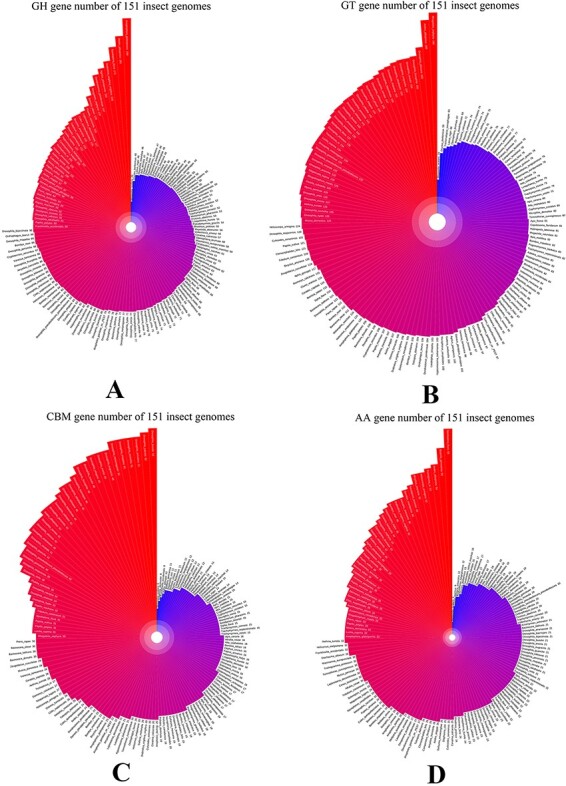
*GH*, *GT*, *CBM* and *AA* gene family distribution of 151 insect genomes in data summary part of iCAZyGFADB.

ICAZyGFADB database contains 11 parts. They are respectively Home, CAZyme, Tools, Download, Links, Help, Register/Login, Data summary, Search, Submit and What’s New& Cite us (Figure 3). On the Home page, it describes which gene function annotation tools this database contains, completes annotation analysis of the *CAZyme* gene family of 151 insect genomes, is user-friendly, and is completely free for users to use. The CAZyme column is the CAZyme annotation toolbar, showing how to use the CAZyme annotation tool to annotate and analyze the function of the *CAZyme* gene family for all protein sequences in insect genomes. In the Tools bar, there are eight gene function annotation tools and one general annotation tool. In this database, all members of *CAZyme* gene family in 151 insect genomes have been annotated and analyzed. The research results show that there are 35 572 *CAZyme* genes, including 11 732 *GHs*, 15 671 *GTs*, 3635 *CBMs*, 4129 *AAs*, 377 *CEs* and 28 *PLs* in 151 insect genomes. In the Download column, there are links to download the protein sequences and CDS sequences of all members of the *CAZyme* gene family of 151 insect genomes, which are mainly divided into two categories: one is downloaded through species classification, and the other is downloaded through the six categories of *AA, CBM, CE, GH, GT* and *PL* in the *CAZyme* gene family. The Links column contains two contents: Genome Resource and Genome Annotation. Genome Resource lists the genome databases involved in this database, including NCBI, Ensembl, DDBJ, InsectBase and so on, and Genome Annotation lists genome annotation databases used by gene function annotation tools involved in this database, such as dbCAN meta server, GO, KEGG, COG, KOG, SwissProt, Pfam and so on. The Help column is the user manual of the database website. The Register/Login column provides the buttons that users need to register an account and log in an account before using the database. In the column of Data summary, the number distribution of six gene families (*AA, CBM, CE, GH, GT* and *PL*) in 151 insect genomes is shown by rose chart and histogram and the distribution of insect numbers per order for 151 insects is shown by sunflower chart (Figure 1). Among the 151 insect genomes, the number of *GH* gene families ranges from 20 to 198, of which the largest is *Anoplophora glabripennis* and the smallest is *Megaselia scalaris* (Figure 4A); that of *GT* gene families ranges from 33 to 196, of which the largest is *Rhagoletis zephyria* and the smallest is *M. scalaris* (Figure 4B); that of *CBM* gene families ranges from 6 to 53, of which the largest is *Drosophila busckii* and the smallest is *M. scalaris* (Figure 4C); that of *AA* gene families ranges from 4 to 65, of which the largest is *Spodoptera litura* and the smallest is *M. scalaris* (Figure 4D); that of *CE* gene families ranges from 0 to 16 (Supplementary Figure S1); that of *PL* gene families ranges from 0 to 11, moreover, most of the 151 insect genomes have no *PL* gene family members (Supplementary Figure S2). The Search column mainly provides two search functions, one is direct search, and the other is advanced search. Both types of searches are classified from gene and gene family. Advanced retrieval can select a single insect species or all insect species of a single order for classified retrieval (Supplementary Figure S3). The Submit toolbar provides a window for users to upload the annotation result data of *CAZyme* gene family for their own research on insect genome to this database for sharing. What’s New& Cite us section describes the version information published by the database and the reference method when users need to reference the data and annotation tools published by this database.

### Gene function annotation tools of iCAZyGFADB and its usage method

In order to integrate multiple gene function annotation tools, reduce the cost of genome functional gene annotation and improve the convenience of functional gene annotation, we have developed iCAZyGFADB, which is a gene function annotation database for insect genomes. iCAZyGFADB has nine gene function annotation tools, including CAZyme, GO, KEGG, COG, eggNOG, SwissProt, Pfam, KOG and AnimalTFDB annotation tools. CAZyme annotation tool is the most important tool in this database. It can annotate all protein sequences of any insect genome to all members of the *CAZyme* gene family. It lays a theoretical foundation for further study on the molecular function of *CAZyme* gene family in insect genome. It provides some data analysis support for the molecular mechanism of lignocellulose degradation by phytophagous insects. GO, KEGG, COG, eggNOG, SwissProt, Pfam, KOG and AnimalTFDB annotation tools in this database are common and necessary annotation tools for functional gene annotation in the genome. They are also indispensable annotation tools for better studying the distribution and specific functions of functional genes in the genome.

**Figure 5.**
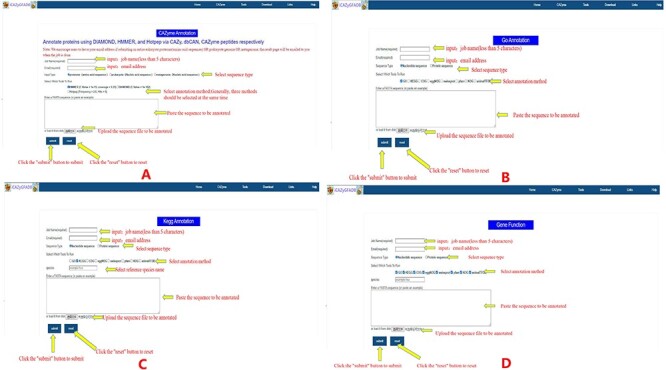
Instructions for the use of nine gene function annotation tools such as CAZyme, GO, KEGG and all annotation tools.

The CAZyme annotation tool is used in the following ways: first, input the name of the running Job and the E-mail to accept the running results; second, select input data type and the annotation running method; third, copy or upload the FASTA format file of all protein sequences in the insect genome to be run; fourth, click the submit button to start running (Figure 5A). The other eight gene function annotation tools are used as follows: the first step is to select the annotation tool by clicking the annotation tool name in the Tools toolbar, Step 2, enter the annotation tool interface and enter the corresponding parameters, Step 3, input the name of the running Job and the E-mail to accept the running results, select input data type and the annotation running method. Step 4, copy or upload the FASTA format file of all protein sequences in the insect genome to be run and click the submit button to start running (Figure 5B and D). It is worth noting that when using the KEGG annotation tool to annotate gene functions, it is necessary to select an additional name abbreviation parameter of the reference species (Figure 5C). The results of the nine gene function annotation tools in this database are sent to the filled mailbox in the form of e-mail. Users can download the running results after opening the result connection received through the mailbox. The time required to annotate an insect genome by these nine gene function annotation tools varies, generally ranging from tens of minutes to several hours, and not more than 24 hours at most. It should be noted that this database has a tool that can run eight gene function annotation tools at one time. If eight annotation tools are running for all protein sequences of an insect genome at the same time, it may take 24–48 hours to complete. The larger the insect genome, the more genes it encodes, that is, the more protein sequences it contains, and the longer the time required for gene functional annotation.

### Advantages and applications of gene function annotation tools in iCAZyGFADB

The CAZyme annotation tool in this database can annotate and analyze the *CAZyme* gene family in all protein sequences of any insect genome. Generally, the size of all protein sequence files annotated of insect genomes is not >30 M, and most of them are between 10 and 20 M. When the CAZyme annotation tool is used to annotate insect protein sequence files of size >50 M, it is necessary to separate it into two files for annotation, because the size of the protein sequence files annotated by this annotation tool cannot exceed 50 M. This is one of the advantages of this database, as currently, many public and free web version databases cannot annotate the functionality of entire species with protein sequence files exceeding 20 M. The CAZyme annotation tool usually takes tens of minutes to several hours to run the results. Similarly, GO, KEGG, COG, eggNOG, SwissProt, Pfam, KOG and AnimalTFDB annotation tools also usually take tens of minutes to several hours to run the results. The nine gene function annotation tools in this database are very good in terms of annotation efficiency and running speed. Compared with other single gene function annotation tools (such as GO, KEGG, COG, eggNOG, SwissProt, Pfam, KOG and AnimalTFDB) or cloud platforms containing multiple gene annotation tools, this database has the following three advantages. First, this database has the advantage of integrating nine gene function annotation tools. Using a single public database such as Pfam, GenBank and UniProt alone to annotate the gene function of the genome is time-consuming and cumbersome. The gene functions of each database annotated in this database are the most comprehensive, and it takes less time to annotate the gene functions of nine databases at once than the total time spent using nine individual database annotations alone. Second, this database can annotate all protein sequences of any insect genome using eight gene annotation tools at the same time and the annotation results can be produced within the time range acceptable to the userand correctly meet the requirements of biological genome function research. Third, this database is user-friendly and completely free for users to use. For example, as of 1 September 2022, the NCBI database has published 1802 insect genomes. There are insect genomes’ sequences of 292 species that have been annotated into protein sequences. The protein sequences of these 292 insect genomes can be used for annotation analysis of nine gene function annotation tools in this database. The other 1510 insect genome sequences need to be annotated by the genome annotation tool for annotation analysis of nine gene annotation tools in this database. It can be seen that a better biological information analysis platform is needed for the huge insect genome function research and gene function analysis. As it happens, iCAZyGFADB, the data platform we developed, just meets the needs of insect genome researchers and provides theoretical data support for gene function annotation research and biological big data.

## Discussion

### *CAZyme* gene annotation function

Compared with the CAZyme annotation database (dbCAN2 metaserver) developed by Zhang *et al.* (11) to analyze a *CAZyme* gene family of the genomes, the size of submitted protein sequence files to be annotated and analyzed in our database CAZyme annotation (iCAZyGFADB) is much larger, up to 50 M. However, the size of submitted protein sequence files used in the CAZyme annotation database designed by Zhang *et al.* (11) cannot allow exceed 20 M, and the protein file with the size exceeding 20 M needs to be split to be annotated. Our database saves the trouble of splitting, and the annotation speed is faster.

### Advantages of gene function annotation tools of iCAZyGFADB

In addition to completing the annotation of a *CAZyme* gene family in 151 insect genomes, iCAZyGFADB also designed eight gene function annotation tools that can quickly annotate gene family functions in the genome. GO, KEGG, COG, eggNOG, SwissProt, Pfam, KOG and AnimalTFDB annotation tools (4–11, 13) of iCAZyGFADB can carry out independent annotation analysis on the protein sequences of each species. Each tool has a fast annotation speed and takes a short time, generally within tens of minutes to 48 h. The fastest one takes tens of minutes, and the slowest one takes only 2 days. The eight gene annotation tools of iCAZyGFADB can annotate the protein sequence of a species for eight functions at the same time, and all the time will not exceed 48 h. Moreover, compared with the annotation by a single software package or website of the eight gene annotation tools, the eight gene annotation tools integrated in our database (iCAZyGFADB) have no difference in the speed, time and accuracy of annotation. The eight gene annotation tools in our database (iCAZyGFADB) have another advantage, that is, they can annotate the genome of a species at the same time with eight tools at one time, which is not available in a single software package or website of the eight tools. Some of the single software package or websites of the eight annotation tools require programming language to operate when annotating genome functions, and the eight annotation tools integrated by our website completely solve this problem.

The resultant data annotated by nine gene annotation tools, such as GO, KEGG, KOG and COG, included in a iCAZyGFADB database are presented in table form. The result files can be downloaded and organized. Through the statistical analysis of the parameter range of *e*-values, it is found that the results obtained are not different from those obtained by using single databases such as GO, KEGG, etc.

Our database, iCAZyGFADB, has two major advantages. First, it is free and friendly to users. No matter in the academic or business circles, there is no biological data analysis website with nine gene function annotations at the same time, and it is free and open to users. Our database just meets this academic demand.

## Supplementary Material

baad086_Supp

## Data Availability

All data are incorporated into the article and its online supplementary material.

## Declarations

Ethics approval and consent to participate. Not applicable because this article does not contain any studies with human or animal subjects.
