# Supplementary figures and images for: iCAZyGFADB: an insect CAZyme and gene function annotation database
Source: Database (Oxford). 2023 Nov 28;2023:baad086. doi: 10.1093/database/baad086 (PMC10684042; doi:10.1093/database/baad086)

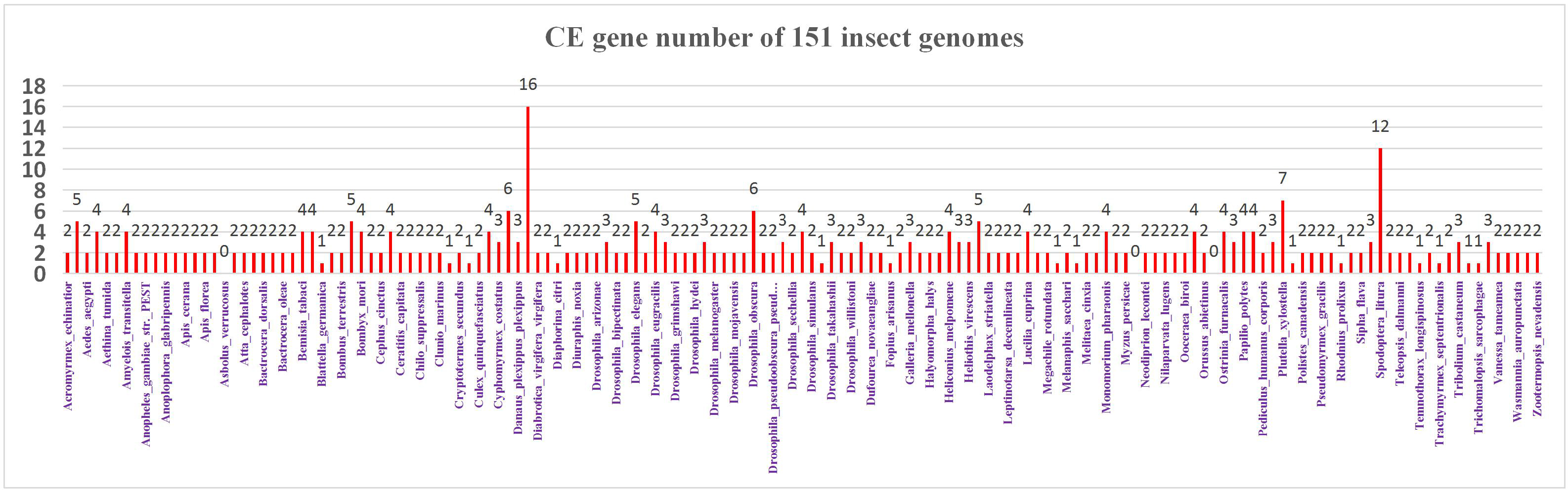

Supplement: baad086_Supp [file baad086_supp.zip › suppl_data/Supplementary Fig1.jpg]

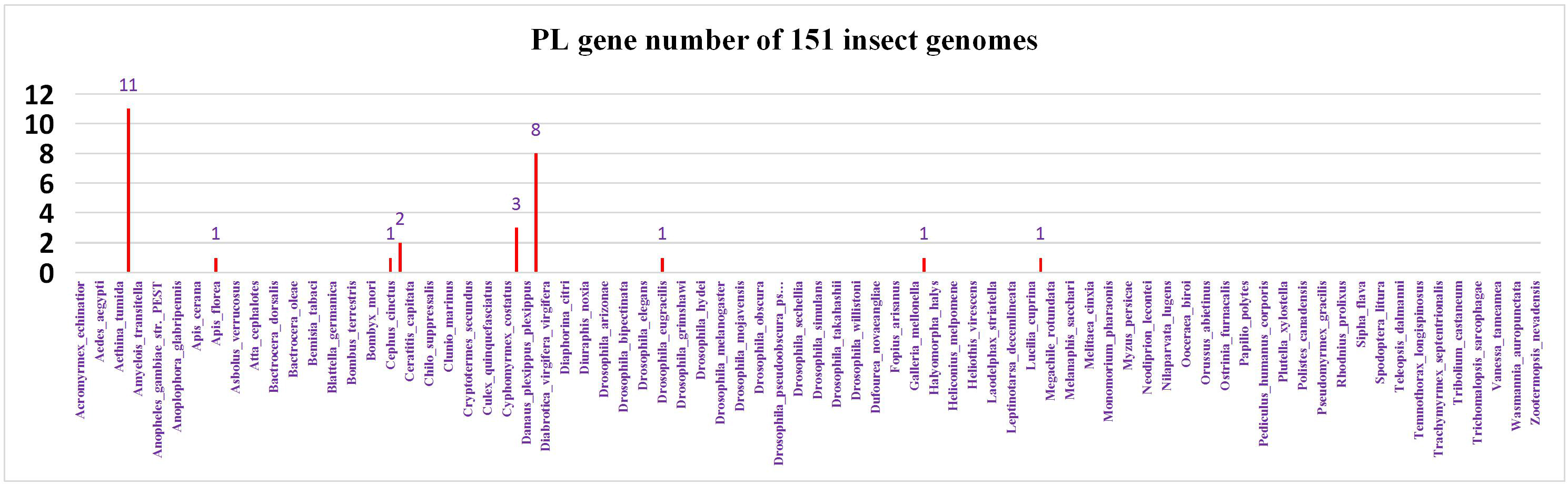

Supplement: baad086_Supp [file baad086_supp.zip › suppl_data/Supplementary Fig2.jpg]

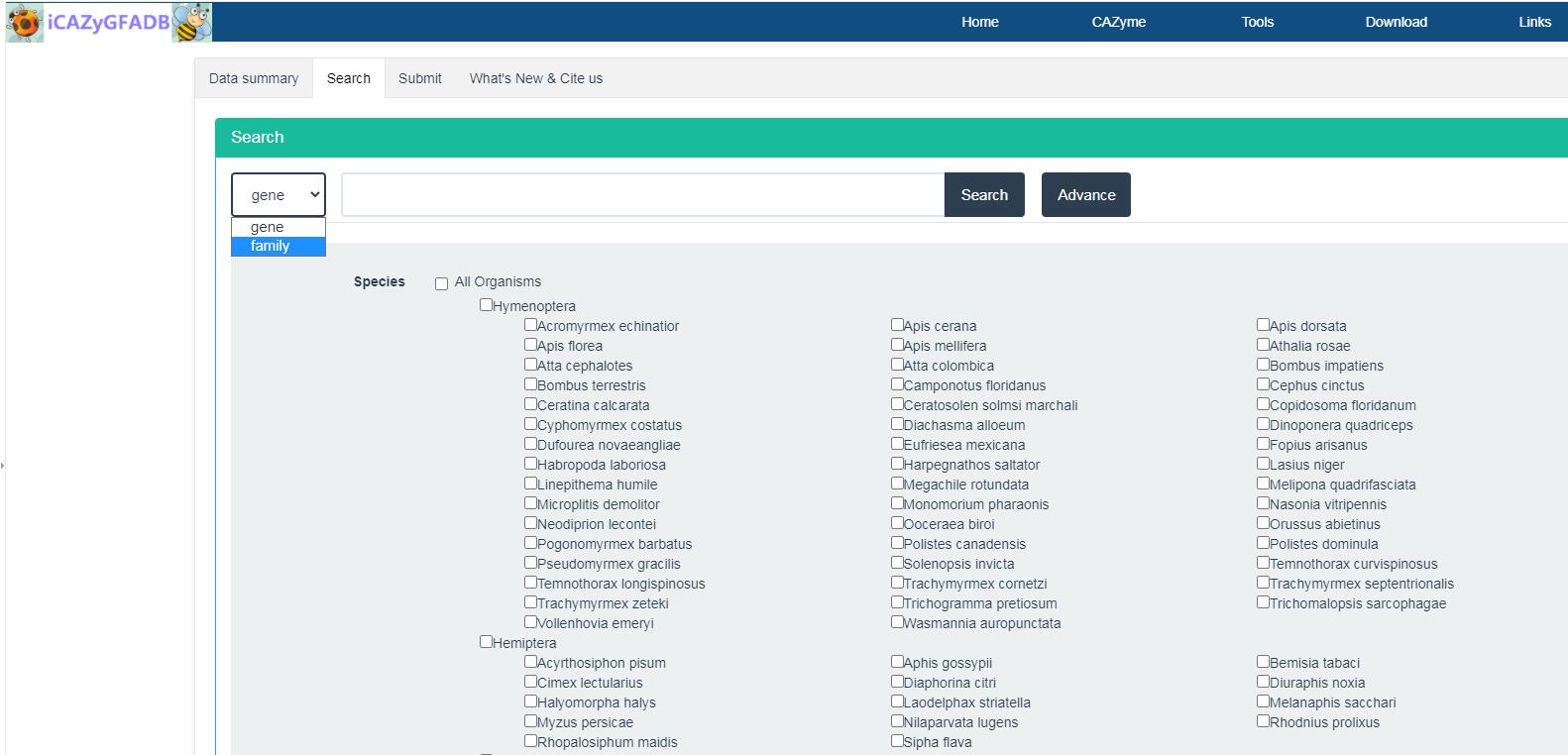

Supplement: baad086_Supp [file baad086_supp.zip › suppl_data/Supplementary Fig3.jpg]
